# Self-Interaction of Human Pex11pβ during Peroxisomal Growth and Division

**DOI:** 10.1371/journal.pone.0053424

**Published:** 2013-01-07

**Authors:** Nina A. Bonekamp, Sandra Grille, Maria Joao Cardoso, Monica Almeida, Miguel Aroso, Silvia Gomes, Ana Cristina Magalhaes, Daniela Ribeiro, Markus Islinger, Michael Schrader

**Affiliations:** 1 Centre for Cell Biology and Department of Biology, University of Aveiro, Campus Universitário de Santiago, Aveiro, Portugal; 2 College of Life and Environmental Sciences, Biosciences, University of Exeter, Exeter, Devon, United Kingdom; Klinikum rechts der Isar der TU München, Germany

## Abstract

Pex11 proteins are involved in membrane elongation and division processes associated with the multiplication of peroxisomes. Human Pex11pβ has recently been linked to a new disorder affecting peroxisome morphology and dynamics. Here, we have analyzed the exact membrane topology of Pex11pβ. Studies with an epitope-specific antibody and protease protection assays show that Pex11pβ is an integral membrane protein with two transmembrane domains flanking an internal region exposed to the peroxisomal matrix and N- and C-termini facing the cytosol. A glycine-rich internal region within Pex11pβ is dispensable for peroxisome membrane elongation and division. However, we demonstrate that an amphipathic helix (Helix 2) within the first N-terminal 40 amino acids is crucial for membrane elongation and self-interaction of Pex11pβ. Interestingly, we find that Pex11pβ self-interaction strongly depends on the detergent used for solubilization. We also show that N-terminal cysteines are not essential for membrane elongation, and that putative N-terminal phosphorylation sites are dispensable for Pex11pβ function. We propose that self-interaction of Pex11pβ regulates its membrane deforming activity in conjunction with membrane lipids.

## Introduction

Peroxisomes are single-membrane bound, multifunctional and highly dynamic organelles of most eukaryotic cells, which fulfil important metabolic functions in hydrogen peroxide and lipid metabolism. Their function has also been linked to developmental processes, stress response, age-related disorders, and antiviral innate immunity [1], [2], [3], [4], [5]. Remarkably, the peroxisomal compartment shows high plasticity and responds to developmental, environmental, and metabolic stimuli with alterations in organelle number, morphology and protein content. Peroxisomes can multiply (or proliferate) by growth and division of pre-existing organelles (reviewed in [6]) or, as particularly demonstrated in yeast, can form *de novo* from the endoplasmic reticulum (ER) [7], [8], [9]. Whereas considerable progress has been made in the identification of key factors involved in these processes, the underlying mechanisms and the regulation of these processes are only poorly understood. The assembly of peroxisomes and protein import into the organelle requires the action of essential proteins, so called peroxins, which are encoded by *PEX* genes. Mutations in many *PEX* genes have been identified as the cause of severe and often lethal peroxisome biogenesis disorders (e.g. Zellweger syndrome) [10], [11].

Peroxisome formation by growth and division involves the deformation and elongation of the peroxisomal membrane, its constriction and final scission [12], [13]. Similar to *de novo* biogenesis from the ER, growth and division of peroxisomes follows a multistep maturation pathway, which results in the formation of new daughter peroxisomes [14], [15]. In mammals, Pex11 proteins are so far the only proteins discovered capable of deforming and elongating the peroxisomal membrane [6], [16]. Hence, the mechanistic details of peroxisomal growth and division and the individual functions of the human Pex11 proteins have attracted great attention as they have been linked to new disorders affecting peroxisome morphology and dynamics [17], [18].

It has recently been reported that Pex11 proteins feature amphipathic helices that can insert into the peroxisomal membrane, thus influencing membrane bending [19], [20]. In line with this, Pex11 proteins are suggested to reorganize the peroxisomal membrane prior to fission [21], [22], [23] and to mediate interactions with the peroxisomal fission machinery [20], [24], [25]. The machinery for membrane scission includes the membrane adaptor proteins Fis1 and Mff, which are involved in the recruitment of the dynamin-like large GTPase DLP1/Drp1 to constriction sites on the peroxisomal membrane [24], [26], [27], [28], [29]. DLP1 is supposed to assemble in spiral-like structures around constricted membranes to mediate membrane scission through GTP hydrolysis leading to the formation of new peroxisomes. Interestingly, mitochondria and peroxisomes, which are metabolically linked to each other, share these key components of their division machinery supporting a closer interorganellar relationship [6], [30], [31], whereas Pex11 proteins are exclusively peroxisomal.

Pex11 proteins are conserved amongst species; however, many organisms contain various “isoforms” which are poorly characterized on a functional level, and may differ in their biochemical properties. Furthermore, their membrane topology is not entirely clear and may vary amongst different species. The mammalian genome encodes for three Pex11 proteins, Pex11pα, Pex11pβ, and Pex11pγ, which are thought to be integral membrane proteins with their N- and C-termini facing the cytosol [16], [20], [21]. Pex11pβ is broadly expressed in mammalian tissues, whereas Pex11pα and Pex11pγ expression is tissue-specific. Studies on knock-out mice revealed that a loss of Pex11pα can be tolerated, with no obvious effect on peroxisome number or metabolism [32], whereas knock-out of Pex11pβ causes neonatal lethality and is accompanied by several defects reminiscent of Zellweger syndrome [33]. Very recently, the first patient with a defect in peroxisome division based on a homozygous non-sense mutation in the *PEX*11β gene was identified [18]. In contrast to the severe clinical phenotype of the Pex11pβ knock-out mice, the patient presented a milder phenotype with normal biochemical parameters of peroxisomes including, however, congenital cataracts, mild intellectual disability, progressive hearing loss, sensory nerve involvement, gastrointestinal problems and recurrent migraine-like episodes.

Here, we addressed the exact membrane topology of *Hs*Pex11pβ, and demonstrated that *Hs*Pex11pβ is an integral transmembrane protein with two transmembrane domains flanking an internal region exposed to the peroxisomal matrix. Based on the topology data, we characterized functional motifs including potential phosphorylation sites and cysteine residues in its N-terminal domain. We demonstrate that a previously uncharacterized amphipathic helix (Helix 2) is essential for peroxisome membrane elongation and self-interaction of *Hs*Pex11pβ. Furthermore, we show that complex formation of *Hs*Pex11pβ strongly depends on the detergent used for solubilization. We propose that self-interaction of *Hs*Pex11pβ regulates its membrane deforming activity.

## Materials and Methods

### Plasmids

Plasmids encoding for *Hs*Pex11pα-Myc, *Hs*Pex11pβ-Myc, Myc-*Hs*Pex11pα, Myc-*Hs*Pex11pβ, Myc-*Hs*Pex11pγ, YFP-*Hs*Pex11pβ and *HsPex11pβ*-YFP were described before [14], [21], [28], [34]. The following *Hs*Pex11pβ deletion constructs were generated by subcloning: N-terminal deletions Pex11pβΔN40-Myc, Pex11pβΔN60-Myc, and Pex11pβΔN70-Myc; Myc-Pex11pβΔGly is missing a glycine-rich region (Δaa159–182; gsggvpggsetgglggpgtpggg) (**Table S1**). Mutations were introduced through site-directed mutagenesis via PCR with oligonucleotide pairs harbouring the respective mutation (**Table S1**). The following plasmids were generated: phospho-mimicking mutants Pex11pβ-Myc^S11A^, Pex11pβ-Myc^S11D^, Pex11pβ-Myc^S38A^ and Pex11pβ-Myc^S38D^; cysteine mutants Pex11pβ-Myc^C18S^, Pex11pβ-Myc^C25S^, Pex11pβ-Myc^C85S^, Pex11pβ-Myc^C18S-C25S^ and Pex11pβ-Myc^C18S-C25S-C85S^ (**Table S1**). Plasmid Pex11pβ-Myc^A21P^ was obtained by cloning the coding sequence of Pex11pβ-Myc^A21P^ (synthesized by Eurofins MWG, Ebersberg, Germany) into pcDNA3 (Invitrogen, Life Technologies, Grand Island, NY, USA) (**Table S1**). In-frame insertion and mutations of all constructs were verified by sequencing (Eurofins MWG, Ebersberg, Germany). For a schematic overview of all plasmids used, see **Fig. S1**.

### *In silico* Analysis

Potential transmembrane domains were predicted using SOSUI (http://bp.nuap.Nagoya-u.ac.jp/sosui/); Toppred tool, Mobyle@Pasteur (http://mobyle.pasteur.fr/cgi-bin/portal.py#welcome); HMMTOP (http://www.enzim.hu/hmmtop/); TMPred (http://www.ch.embnet.org/software/TMPRED_form.html); TMHMM Server, v 2.0 (http://www.cbs.dtu.dk/services/TMHMM/); PredictProtein (http://www.predictprotein.org/); Split 4.0 server (http://split.pmfst.hr/split/4/) (**Fig. S2B**). Protein fragment size after proteinase K digest was calculated using PeptideMass (http://web.expasy.org/peptide_mass/).

Potential phosphorylation sites or potential binding sites for kinases were predicted by KinasePhos 2.0 (http://kinasephos2.mbc.nctu.edu.tw/index.html); NetPhos 2.0 (http://www. cbs.dtu.dk/services/NetPhos/); DISPHOS (http://core.ist.temple.edu/pred/pred.html); NetPhosK (http://www.cbs.dtu.dk/services/NetPhosK/), Scansite MotifScan (http://scansite.mit.edu/motifscan_seq.phtml); ScanProsite (http://expasy.org/tools/scanprosite/) and ELM (http://elm.eu. org/). Alignment of Pex11β protein sequences from different species was performed using the ClustalW2 tool (http://www.ebi.ac.uk/Tools/msa/clustalw2/) (**Fig. S3**).

### Antibodies

Rabbit polyclonal antibodies were used as follows: anti-GFP (Invitrogen, Life Technologies, Grand Island, NY, USA), anti-acyl-CoA oxidase (AOX), anti-PMP70 [35] (kindly provided by A. Völkl, University of Heidelberg, Germany), anti-*Hs*Pex11pβ (ab74507, Abcam Inc., Cambridge, UK) (see our Abcam Abreview) and anti-Pex14p (a kind gift from D. Crane, Griffith University, Brisbane, Australia). The following mouse monoclonal antibodies were used: anti-Pex19p, (purchased from BD Transduction Laboratories, San Diego, CA, USA) and anti-Myc epitope 9E10 (Santa Cruz Biotechnology, Santa Cruz, CA, USA). Species-specific anti-IgG antibodies conjugated to HRP or to the fluorophores TRITC and Alexa 488 were obtained from BioRad (Hercules, CA, USA), Dianova (Heidelberg, Germany), Molecular Probes Europe (Leiden, The Netherlands) and Invitrogen (Life Technologies, Grand Island, NY, USA).

### Cell Culture, Transfection and Microscopy

COS-7 cells (ATCC CRL-1651) were maintained in DMEM supplemented with 100 units/ml penicillin, 100 µg/ml streptomycin and 10% FCS (PAA Laboratories GmbH, Cölbe, Germany) at 37°C in a 5% CO_2_-humidified incubator. For some experiments, cells were transferred to lipid-free Panserin™ 401 medium, a serum-free nutrient mixture (kindly provided by Biotech GmbH, Aidenbach, Germany). Cells were transfected with polyethylenimine (25 kDa PEI, Sigma-Aldrich, St. Louis, MO, USA) or by electroporation using the ECM 630 Electro Cell Manipulator (BTX Harvard Apparatus, Holliston, MA, USA) [21], [27]. For immunofluorescence microscopy, cells were fixed with 4% para-formaldehyde in PBS (20 min), permeabilized with either 0.2% Triton X-100 (10 min), 2.5 µg/ml digitonin (5 min) or methanol (-20°C, 6 min), blocked with 1% BSA solution (15 min) and incubated with the indicated primary and secondary antibodies (1 h each). Cells were mounted in Mowiol 4–88 containing n-propylgallate as anti-fading as described [21], [34]. Samples were analysed using an Olympus IX81 microscope (Olympus Optical Co. GmbH, Hamburg, Germany) equipped with the appropriate filter combinations and a 100×objective (Plan-Neofluar, 100x/1.35 oil objective). Images were acquired with an F-view II CCD camera (Soft Imaging System GmbH, Münster, Germany) driven by Soft Imaging software. Digital images were optimized for contrast and brightness using Adobe Photoshop software (Adobe Systems, San Jose, CA, USA). For quantitative analysis of peroxisome morphology, 100–200 cells per coverslip were examined blind and categorized as cells with spherical (0.1–0.3 µm) or elongated (2–5 µm in length) peroxisomes as described [36]. Usually 2–3 coverslips per preparation were analyzed and three independent experiments were performed. Significant differences between experimental groups were detected by analysis of variance for unpaired variables using Microsoft Excel software. Data are presented as means ± S.D., with an unpaired *t* test used to determine statistical differences. *p* values <0.05 were considered as significant, and *p* values <0.01 were considered as highly significant.

### Sample Preparation, Gel Electrophoresis and Immunoblotting

COS-7 cells transfected with Pex11pβ constructs were fixed with 4% para-formaldehyde, washed with PBS and treated with Triton X-100 (see above). The resulting supernatants were collected and cleared by centrifugation (16,200×g, 15 min). The remaining cells (and non-treated controls) were rinsed with PBS and centrifuged (800×g, 5 min, 4°C). Cell pellets were then lysed (25 mM Tris-HCl, pH 8.0, 50 mM sodium chloride, 0.5% sodium deoxycholate, 0.5% Triton X-100 and a protease-inhibitor mix). The samples were passed ten times through a 26-gauge syringe needle, incubated by mixing at 4°C for 30 min and cleared by centrifugation (16,200×g, 15 min). Protein concentrations were determined using the Bradford assay (BioRad, Hercules, CA, USA) and proteins were precipitated with TCA. Equal amounts of protein were separated by SDS-PAGE (10–15% PAA gels) under reducing and non-reducing conditions, transferred to nitrocellulose (Schleicher and Schüll, Dassel, Germany) by semi-dry transfer (BioRad, Hercules, CA, USA) and analysed by immunoblotting. Immunoblots were processed using HRP-conjugated secondary antibodies and enhanced chemiluminescence reagents (GE Healthcare, Waukesha, WI, USA).

### Proteinase K digest and Carbonate Extraction

Peroxisome-enriched fractions were prepared from COS-7 cells transfected with the indicated constructs and from controls (four 100 mm cell culture dishes) by differential centrifugation as described [37], [27]. Briefly, cells were harvested 48 h after transfection, resuspended in peroxisome homogenization buffer (20 mM MOPS-KOH pH 7.4, 250 mM sucrose, 1 mM EDTA-NaOH pH 7.4, protease inhibitor mix) and homogenized using a 1 ml syringe equipped with a 26 G needle. The resulting homogenate was centrifuged at 500×g (5 min, 4°C) to remove cellular debris. The pellet was re-homogenized and the supernatants were pooled and centrifuged at 2,000×g (10 min, 4°C). The resulting supernatant was then centrifuged at 25,000×g (25 min, 4°C) (Beckmann Avanti-J251, Beckman Coulter Inc., Indianapolis, IN, USA) to enrich for peroxisomes. The final pellet was resuspended in 100 µl of peroxisome homogenization buffer. Sixty µg of samples and appropriate controls were incubated with 25 µl of proteinase K (from a 2 mg/ml stock in 20 mM MOPS-KOH, pH 7.4) in the presence or absence of 1% Triton X-100. As an alternative to Triton X-100 permeabilization, peroxisomal membranes were ruptured by sonication (3 times for 10 sec, 100 W, on ice). Proteinase K digest was carried out for 30–45 min on ice and then stopped by the addition of PMSF (5 mM final concentration). All samples were brought to a volume of 100 µl with homogenization buffer and precipitated by TCA.

For carbonate extraction, peroxisome-enriched fractions were resuspended in ice-cold carbonate buffer (100 mM Na_2_CO_3,_ pH 11.5). Samples and controls were incubated for 30 minutes on ice with gentle shaking every 5 minutes and centrifuged at 223,000×g (1 h, 4°C) in an Optima LE-80K Ultracentrifuge (Ti 80 rotor; Beckman Coulter Inc., Indianapolis, IN,). The supernatant was collected and the final membrane pellet was resuspended in peroxisome homogenization buffer. Protein concentrations of all fractions were determined before SDS-PAGE and immunoblotting.

### Sedimentation Analysis

Twenty four hours after transfection, COS-7 cells expressing Pex11pβ-Myc were treated for 30 min at room temperature with 1 mM dithiobis[succinimidyl propionate] (DSP; Pierce, Rockford, IL). After quenching with 50 mM Tris-HCl, pH 7.4 for 15 min at room temperature, cells were harvested, solubilized with Triton X-100 (or digitonin) and lysed as described above. Alternatively, lysates were prepared without previous cross-linking substituting Triton X-100 by 1% digitonin in the lysis buffer. Lysates were applied on top of a 10–47% sucrose step gradient in 50 mM Tris acetate buffer, pH 7.2 with 1 mM EDTA, 0.1% digitonin (Triton X-100) and were centrifuged for 3 h at 125,000×g_av_, 4°C in a Beckman VTi 50 Rotor (Beckman Coulter, Indianapolis, IN, USA). The gradient was eluted into 12 fractions of 1 ml each. For gradient calibration a “Kit for Molecular Weights 14,000–500,000 - Non-denaturing PAGE2” from Sigma-Aldrich (St. Louis, MO, USA) was used. Samples were analyzed by immunoblotting.

## Results

### *Hs*Pex11pβ is a Transmembrane Protein with an Intra-peroxisomal Region and N- and C-termini facing the Cytosol

**Figure 1:**
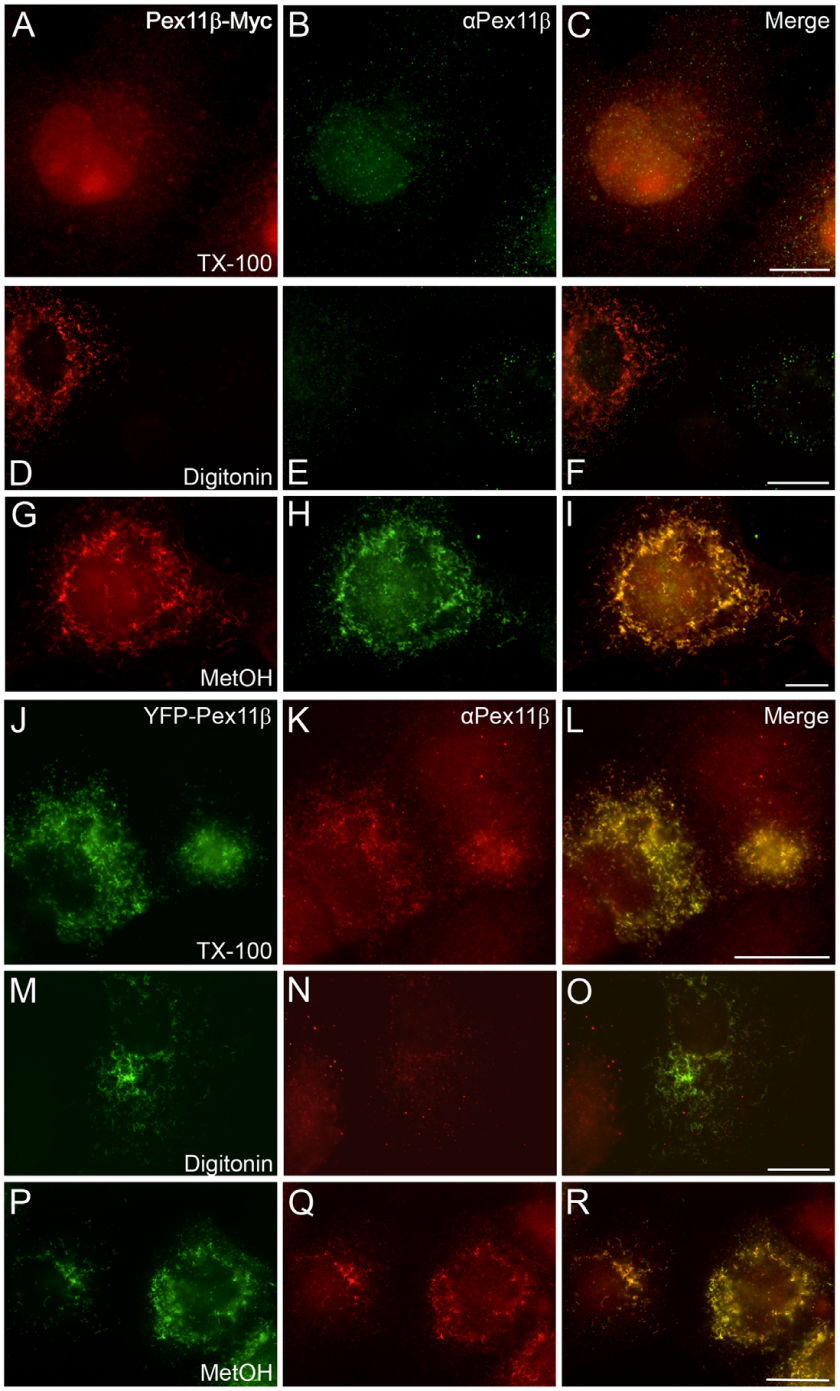
Permeabilization of the peroxisome membrane is required for epitope recognition of the Pex11pβ antibody. COS-7 cells were transfected with Pex11pβ-Myc (**A–I**) or YFP-Pex11pβ (**J–R**) and fixed after 24 hours. Cell membranes were permeabilized with 0.2% Triton X-100 (TX-100) (**A–C, J–L**), 25 µg/ml digitonin (**D–F, M–O**) or methanol (MetOH) (**G–I, P–R**) prior to immunostaining with anti-Myc (**A, D, G**) and anti-Pex11pβ (**middle column)** antibodies. Note that Pex11pβ-Myc is liberated from peroxisomal membranes after postfixation TX-100 treatment (**A–C**), while YFP-Pex11pβ is retained (**J–L**). Bars, 20 µm.

Although Pex11 proteins are conserved amongst most eukaryotic organisms, various isoforms exist which are functionally poorly characterized and may differ in their biochemical properties. Furthermore, their membrane topology is not entirely clear and may vary amongst different species. In contrast to e.g. *Sc*Pex11p [38] or *At*Pex11a [39], the mammalian Pex11 proteins were supposed to be transmembrane proteins with their N- and C-termini exposed to the cytosol [21], [40], [41]. However, *Hs*Pex11pγ was recently reported to dock on the cytosolic site of the peroxisomal membrane [20]. Furthermore, depending on the algorithm used, *in silico* analysis of Pex11pβ does not always result in the prediction of two transmembrane domains as initially proposed [40], [21] (**Fig. S2B**). Determination of the exact topology of Pex11pβ was so far limited by the lack of specific antibodies; however, a newly available antibody directed against an internal site (aa 110–140) corresponding to a region roughly behind the predicted first transmembrane domain enabled further characterization. First, COS-7 cells were transfected with Pex11pβ-Myc and processed for immunofluorescence microscopy by applying antibodies to the Myc-epitope at the very C-terminus and to Pex11pβ itself (**Fig. 1**). When cells were fixed with 4% para-formaldehyde and permeabilized with Triton X-100 (**Fig. 1A–C**), no specific signal for Myc or Pex11pβ was detected which is consistent with our previous observations that Pex11pβ is extracted from peroxisomal membranes after postfixation Triton X-100 treatment [34] (see Figure in “The N-terminal 40 aa of Pex11pβ including Helix 2 are crucial for dimer formation”). In contrast to Triton X-100, postfixation digitonin treatment does not remove Pex11pβ from peroxisomes (see Figure in “The N-terminal 40 aa of Pex11pβ including Helix 2 are crucial for dimer formation”); however, digitonin only permeabilizes the plasma membrane but not peroxisomal membranes [21], [34]. Concordantly, the C-terminal Myc-epitope which is supposed to be exposed to the cytosol [21], is recognized by the Myc antibody (**Fig. 1D–F**) in digitonin-permeabilized cells. No signal corresponding to the Pex11pβ antibody was observed (**Fig. 1E**), indicating that the epitope is not accessible for antibody detection and thus located within the peroxisomal matrix or membrane. Upon combined para-formaldehyde-methanol fixation and membrane permeabilization via methanol, Pex11pβ was readily detected by both the anti-Myc and the anti-Pex11pβ antibodies (**Fig. 1G–I**). Similar observations were made upon overexpression of a YFP-Pex11pβ fusion protein (**Fig. 1J–R**). We recently demonstrated that the addition of a larger protein tag immobilizes Pex11pβ in the peroxisomal membrane, thus rendering it insensitive to postfixation Triton X-100 extraction [34]. As a result, co-localization of YFP and Pex11pβ signals was observed upon membrane permeabilization with either Triton X-100 (**Fig. 1J–L**) or methanol (**Fig. 1P–R**). Consistent with above findings, detection of Pex11pβ with the anti-Pex11pβ antibody upon digitonin permeabilization failed (**Fig. 1M–O**) due to the inaccessibility of the epitope. It should be noted that the N-terminal YFP-tag is accessible after digitonin permeabilization, e.g. with anti-GFP antibodies (not shown). These observations confirm that the new Pex11pβ antibody recognizes an epitope that is only accessible upon permeabilization of the peroxisomal membrane and lies protected within the peroxisomal matrix or membrane.

**Figure 2:**
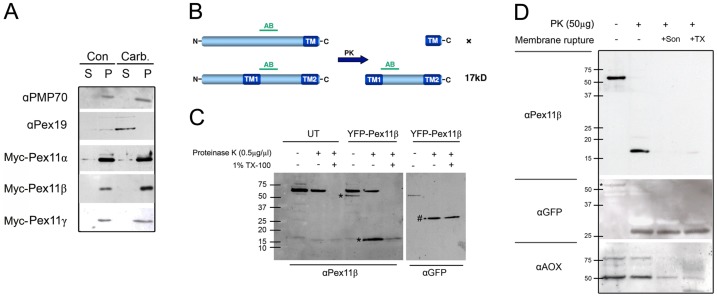
*Hs*Pex11pβ is an integral membrane protein with two transmembrane spans flanking a protease-protected region. (**A**) COS-7 cells were transfected with Myc-tagged Pex11pα, Pex11pβ, or Pex11pγ and subjected to carbonate extraction (Carb.) at pH 11.5 or were mock treated (Con). Equal amounts of protein (P, membrane fraction; S, carbonate extract) were separated by SDS-PAGE on 12.5% acrylamide gels and subjected to immunoblotting with anti-Myc antibodies. PMP70 and Pex19p served as controls for integral and peripheral proteins, respectively. (**B**) Schematic view of potential results of a proteinase K (PK) digest depending on the number and location of putative transmembrane spans within Pex11pβ (see also **Fig. S2**). AB, epitope recognized by anti-Pex11pβ. (**C**) COS-7 cells were transfected with YFP-Pex11pβ or mock transfected (UT). 48 h after transfection, peroxisome-enriched fractions were prepared. Equal amounts of protein were digested with proteinase K in the presence or absence of Triton X-100 (TX-100). Controls were left untreated. Samples were separated by 12.5% SDS-PAGE and immunoblotted using anti-Pex11pβ. As a loading control, the membrane was re-incubated with anti-GFP after membrane stripping. Asterisks indicate the YFP-Pex11pβ band before and after digest. Note that the nonspecific band (approx. 60 kDa) is no longer recognized after repeated use of the Pex11pβ antibody (see **D**). (**D**) As an alternative to (**C**), peroxisome fractions were ruptured by sonication prior to proteinase K digest and immunoblotted as described. As a loading control, the membrane was re-incubated with anti-GFP. Successful membrane rupture was verified by incubation with anti-AOX, a peroxisomal matrix marker.

To re-examine whether Pex11pβ has indeed properties of an integral membrane protein, we performed a carbonate extraction with peroxisome-enriched fractions from COS-7 cells expressing Myc-Pex11pβ as well as the other two Pex11 isoforms, Pex11pα and Pex11pγ. In agreement with previous findings, Pex11pβ was not extractable with sodium carbonate at pH 11.5 and was exclusively detected in the membrane pellet (**Fig. 2A**). Similar results were obtained for Pex11pα and Pex11pγ. The peroxisomal ABC transporter PMP70 served as positive control for an integral peroxisomal membrane protein (**Fig. 2A**). Pex19p, which is partially associated with the peroxisomal membrane via its interaction with Pex3p, served as an example for a peripheral membrane protein, which is sensitive to carbonate extraction (**Fig. 2A**). Our results clearly show that all Pex11p isoforms behave like integral membrane proteins.

### Proteinase K digest of Human Pex11pβ Results in the Formation of a 17 kD Protease-protected Fragment

To further determine the membrane topology of Pex11pβ, a proteinase K digest was performed exploiting the properties of the newly available antibody to Pex11pβ. The location of the epitope (AB in **Fig. 2B**) within the protein provided the following scenarios: if Pex11pβ would only possess one transmembrane domain at its very C-terminus (**Fig. 2B** **, upper panel;** **Fig. S2**), proteinase K digest would result in an almost complete degradation of the protein (and hence the epitope); therefore no signal would be detected. Similarly, the antibody epitope would be digested if a first transmembrane domain at aa 170–200 is assumed (**Fig. S2**). If a first transmembrane domain would be present approximately in the middle of the protein (aa 85–105), the antibody’s epitope would be rendered protease protected, resulting in the formation of a protein fragment of approximately 17 kDa (**Fig. 2B** **, lower panel**). For protease-protection assays, COS-7 cells were transfected with YFP-Pex11pβ. Peroxisome-enriched fractions from non-transfected controls (UT) and transfected cells were mock treated, or incubated with proteinase K in the absence or presence of Triton X-100 (**Fig. 2C**).

After overexpression of YFP-Pex11pβ, the Pex11pβ antibody recognized a corresponding band of approx. 56 kD in the absence of proteinase K, albeit weakly (**Fig. 2C** **, asterisk**). Proteinase K digest resulted in a band shift yielding a fragment of approx. 17 kDa, which was properly recognized by anti-Pex11pβ. Addition of Triton X-100 rendered the fragment sensitive to proteinase K digest (**Fig. 2C** **, asterisks**). The protein fragment was also digested after membrane rupture by sonication (**Fig. 2D**), indicating that this region of Pex11pβ extends into the peroxisomal matrix. In addition, a nonspecific band of around 60 kDa was occasionally detected, which is consistent with the manufacturer’s information.

The N-terminal YFP-tag of YFP-Pex11pβ appeared to be quite resilient to protease action, most probably due to the compact β-barrel structure of GFP and its analogues, and thus served as an excellent loading control after membrane stripping (**Fig. 2C** **, αGFP**). In the absence of proteinase K, the 56 kDa band of YFP-Pex11pβ was detected using an anti-GFP antibody. In the presence of proteinase K, the YFP fusion tag was removed from Pex11pβ and remained unaffected by the action of the protease (**Fig. 2C** **, #**). However, similar band intensities were detected in the absence or presence of Triton X-100, verifying equal loading of lanes. Further incubation of the blotting membranes with anti-AOX antibodies routinely served to ensure integrity of the peroxisomal membrane before addition of Triton X-100 (**Fig. 2D**). The results we obtained after proteinase K digest of Pex11pβ are consistent with a predicted first transmembrane domain located approximately between aa 90–110 (PredictProtein; TM predict [21]; **Fig. S2A**) and a second one between aa 230–255. Thus, Pex11pβ has a major part of its N-terminus exposed to the cytosol and possesses two hydrophobic transmembrane domains flanking an internal region which extends into the peroxisomal matrix or is buried within the lipid bilayer.

Based on the results on Pex11pβ topology provided above, we analyzed putative functional motifs in its sequence and examined their importance for the membrane shaping properties of Pex11pβ.

### A Glycine-rich Region within *Hs*Pex11pβ is Dispensable for Peroxisomal Growth and Division

**Figure 3:**
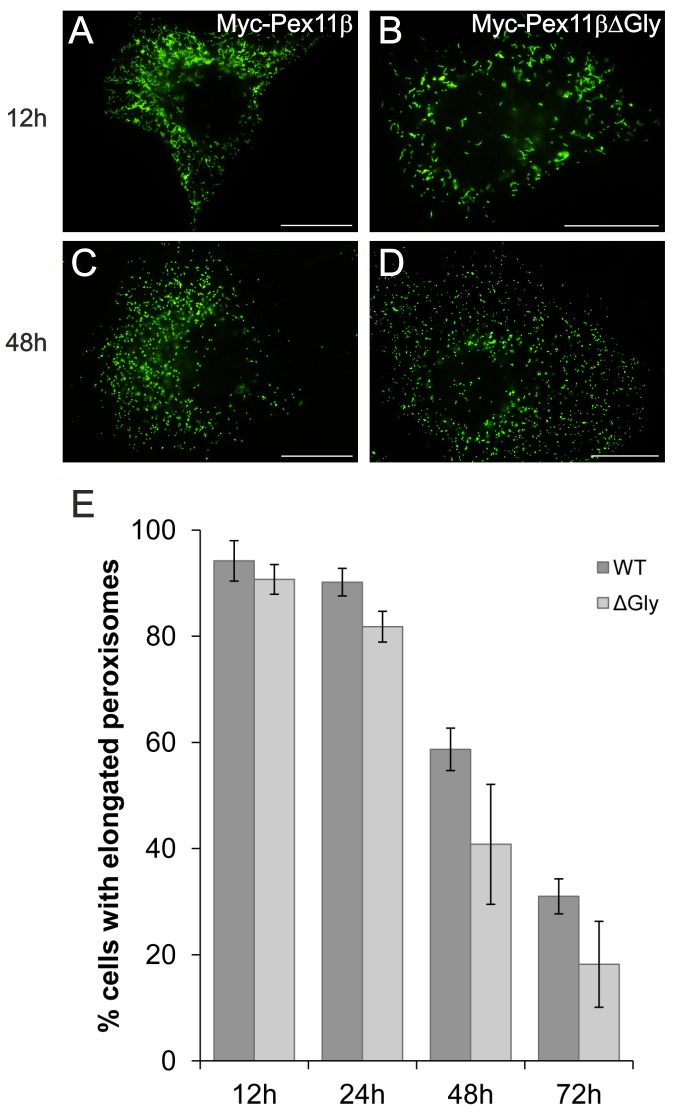
A glycine-rich internal region specific for human Pex11pβ is dispensable for peroxisome elongation and division. COS-7 cells expressing Myc-Pex11pβ (**A, C**) and Myc-Pex11pβΔGly (**B, D**) were processed for immunofluorescence microscopy after 12 and 48 h using anti-Myc (**A–D**). (**E**) Quantitative evaluation of peroxisome morphology over time. Data are from 3 independent experiments and are presented as means ± S.D. Bars, 20 µm.

We observed that human Pex11pβ contains a glycine-rich region at aa positions 159–182 (gsggvpggsetgglggpgtpggg), which is absent in Pex11pα or Pex11pγ. This region is located between the two transmembrane domains and based on our topology studies, is exposed to the peroxisomal matrix (**Fig. S2A**). To examine if the glycine-rich region (which also contains proline residues) is required for Pex11pβ function, we deleted this region resulting in construct Myc-Pex11pβΔGly (**Fig. S1**). Expression in COS-7 cells showed proper targeting to peroxisomes as revealed by immunofluorescence microscopy (**Fig. 3A–D**). Furthermore, deletion of the glycine-rich region had no effect on peroxisome elongation and subsequent division over time when compared to controls expressing wild type Myc-Pex11pβ (**Fig. 3E**). Our data demonstrate that the glycine-rich region within Pex11pβ is dispensable for the targeting to peroxisomes as well as membrane elongation and division.

### Serine Residues S11 and S38 are not Involved in the Regulation of Pex11pβ by Putative Phosphorylation

Only little information is available on the regulation of Pex11 proteins, e.g. by post-translational mechanisms. It has, however, recently been demonstrated in yeast, that *Saccharomyces cerevisiae Sc*Pex11p and *Pichia pastoris Pp*Pex11p are regulated by phosphorylation [42], [43]. Phospho-mimicking “on” and “off” mutants either interfered with peroxisome division giving rise to enlarged and clustered peroxisomes (constitutively dephosphorylated), or resulted in hyperdivision (constitutively phosphorylated).

**Figure 4:**
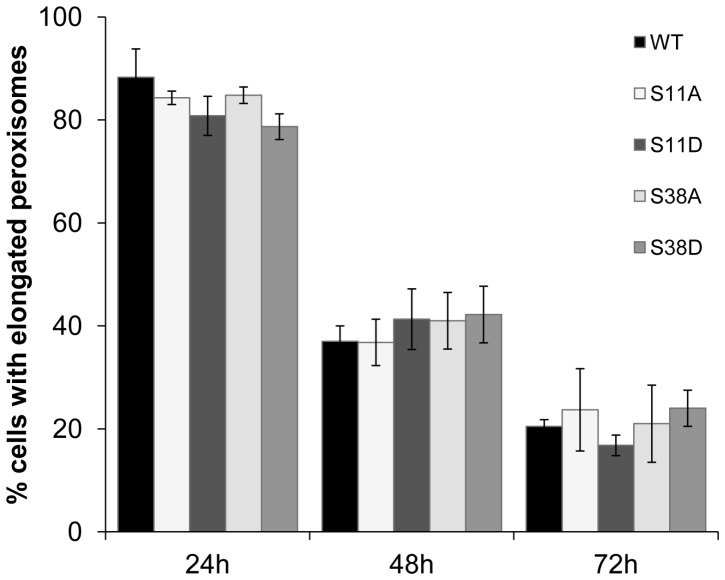
Phospho-mimicking mutants of Pex11pβ do not influence peroxisomal elongation and division. COS-7 cells expressing Pex11pβ-Myc, Pex11pβ-Myc^S11A^, Pex11pβ-Myc^S11D^, Pex11pβ-Myc^S38A^ and Pex11pβ-Myc^S38D^ were processed for immunofluorescence using anti-Myc and anti-Pex14p antibodies (**Suppl. Fig. S4**) and peroxisome morphology was quantified. Data are from 3 independent experiments and are presented as means ± S.D.

To identify potential phosphorylation sites in Pex11pβ, we performed an *in silico* analysis using various prediction tools that either calculate putative phosphorylation sites within the protein or screen for potential kinase binding sites (**Fig. S3A**). The results were combined with a homology screen of various Pex11pβ protein sequences examined for conservation of putative phosphorylation sites (**Fig. S3C**). Several conserved sites were identified at positions S11, S38, S70, S154, S160, S168 and T178 within the human protein, which showed high probability for possible phosphorylation (**Fig. S3B**). Here, we focused on the putative phosphorylation sites S11 and S38 in the 40 aa N-terminal portion of Pex11pβ, because deletion of the 40 aa was sufficient to inhibit membrane elongation and homo-dimerization (see below). Furthermore, our topology results indicate that the residues S11 and S38 are present in the cytosolic portion of Pex11pβ and are thus potentially accessible to cytosolic kinases. Individual point mutations were generated by site-directed mutagenesis. We converted the respective serines to alanine to block putative phosphorylation resulting in constructs Pex11pβ-Myc^S11A^ and Pex11pβ-Myc^S38A^. Furthermore, to generate phospho-mimicking (constitutively phosphorylated) versions we mutated the sequences encoding S11 or S38 to aspartate resulting in constructs Pex11pβ-Myc^S11D^ and Pex11pβ-Myc^S38D^. The constructs were expressed in COS-7 cells and alterations of peroxisome morphology were analyzed at different time points by immunofluorescence microscopy using anti-Myc and Pex14p antibodies (**Fig. S4**). Wild type Pex11pβ induces a prominent elongation of peroxisomes which is followed by division into spherical organelles over time [21], [26] (**Fig. 4** **; Fig. S4**). A similar pattern of morphological alterations was observed in all mutants generated. No enlarged or otherwise altered morphologies were detected and division proceeded normally over time when Pex11pβ-Myc^S11A^ or Pex11pβ-Myc^S38A^ were expressed. Similarly, expression of Pex11pβ-Myc^S11D^ or Pex11pβ-Myc^S38D^ did not result in division at a faster rate. These findings indicate that modifications of S11 and S38 have no impact on peroxisome elongation or division, but do not exclude that other putative phosphorylation sites within Pex11pβ may modulate its activity. So far, we have not obtained any indication that Pex11pβ is phosphorylated under our experimental conditions (e.g. by phospho-labelling). These findings may indicate that yeast and mammalian Pex11 proteins rely on different regulatory mechanisms.

### The Predicted Amphipathic Helix 2 within the First 40 N-terminal aa of Pex11pβ is Required to Elongate the Peroxisomal Membrane

**Figure 5:**
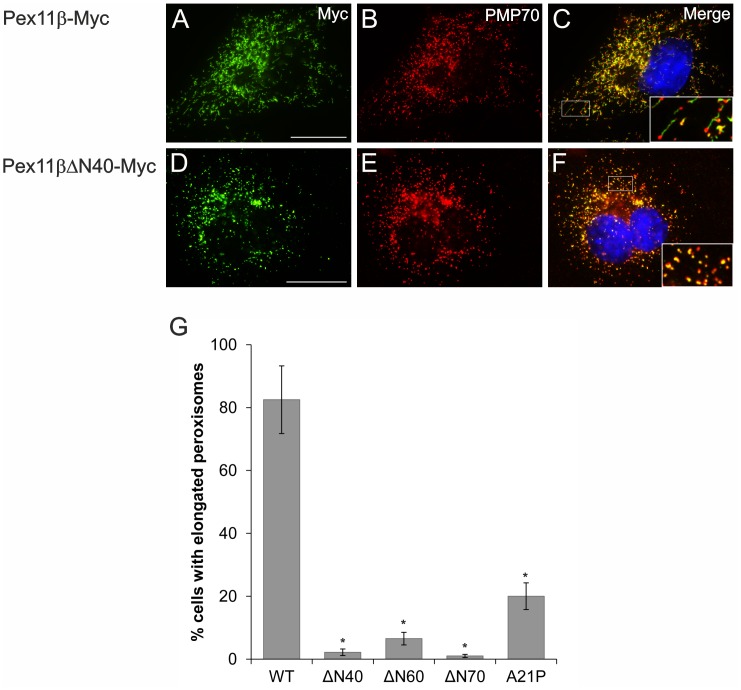
An intact Helix 2 within the first 40 N-terminal aa of Pex11pβ is required to elongate the peroxisomal membrane. COS-7 cells were transfected with Pex11pβ-Myc (**A–C**), the N-terminal deletions Pex11pβΔN40-Myc (**D–F**), Pex11pβΔN60-Myc, Pex11pβΔN70-Myc and the Helix 2-breaking mutant Pex11pβ-Myc^A21P^. Cells were processed for immunofluorescence microscopy after 24 h using anti-Myc (**A, D**) and anti-PMP70 (**B, E**) antibodies. (**G**) Quantitative evaluation of peroxisome morphology. Data are from 3–4 independent experiments and are presented as means ± S.D. (*p<0.01). Bars, 20 µm.

Pex11 proteins possess amphipathic regions which are supposed to play important roles in membrane remodelling and peroxisome proliferation [19]. Pex11pβ contains three potential α-helices (H1-3) within its N-terminal domain (**Figs. S1, S2A**) [19]. Helix 1 is only composed of 6 aa residues, whereas Helix 2 and Helix 3 display larger amphipathic stretches with Helix 3 being the largest one. So far, *in vitro* studies using peptides matching Helix 3 of *Sc*Pex11p, *Hp*Pex11p, and *Hs*Pex11pα and *Hs*Pex11pβ showed the ability of the peptides to elongate negatively charged small unilamellar vesicles [19] suggesting that Helix 3 plays the central role in membrane elongation. To study the potential role of the helices in the regulation of Pex11pβ *in situ*, we generated N-terminally truncated versions (Pex11pβΔN40-Myc, Pex11pβΔN60-Myc, Pex11pβΔN70-Myc) and analyzed their effect on peroxisome morphology (**Fig. 5**). Upon expression in COS-7 cells, all truncated fusion proteins localized to peroxisomes as shown by immunofluorescence and co-localized with the peroxisomal marker PMP70 (**Fig. 5A–F**) (ΔN60, ΔN70 not shown). Interestingly, cells expressing the truncated versions did not exhibit a prominent elongation of peroxisomes (**Fig. 5G** **)**. This is in contrast to the expression of full-length Pex11pβ-Myc, which typically induced a significant membrane elongation (**Fig. 5A–C** **)** [14], [21], [27]. Whereas the ΔN60 and ΔN70 truncations disrupt all helices, the ΔN40 truncation leaves Helix 3 intact (**Figs. S1, S2A**). This indicates that although peptides matching Helix 3 are capable of elongating liposomal structures *in vitro*, also Helix 2 (and region H1) is required for peroxisome elongation in living cells.

To verify this assumption, we introduced a proline at position 21, which breaks the helical structure (Pex11pβ-Myc^A21P^), and analyzed the effect of this version on peroxisome morphology (**Fig. 5G** **)**. Interestingly, expression of this mutant construct did not result in prominent peroxisome elongation, thus confirming the importance of Helix 2 for proper Pex11pβ function.

### ΔN40-Pex11pβ-Myc Fails to Induce Tubular Peroxisomal Accumulations (TPAs) in Conjunction with YFP-Pex11pβ and Shows Altered Membrane Distribution within TPAs

**Figure 6:**
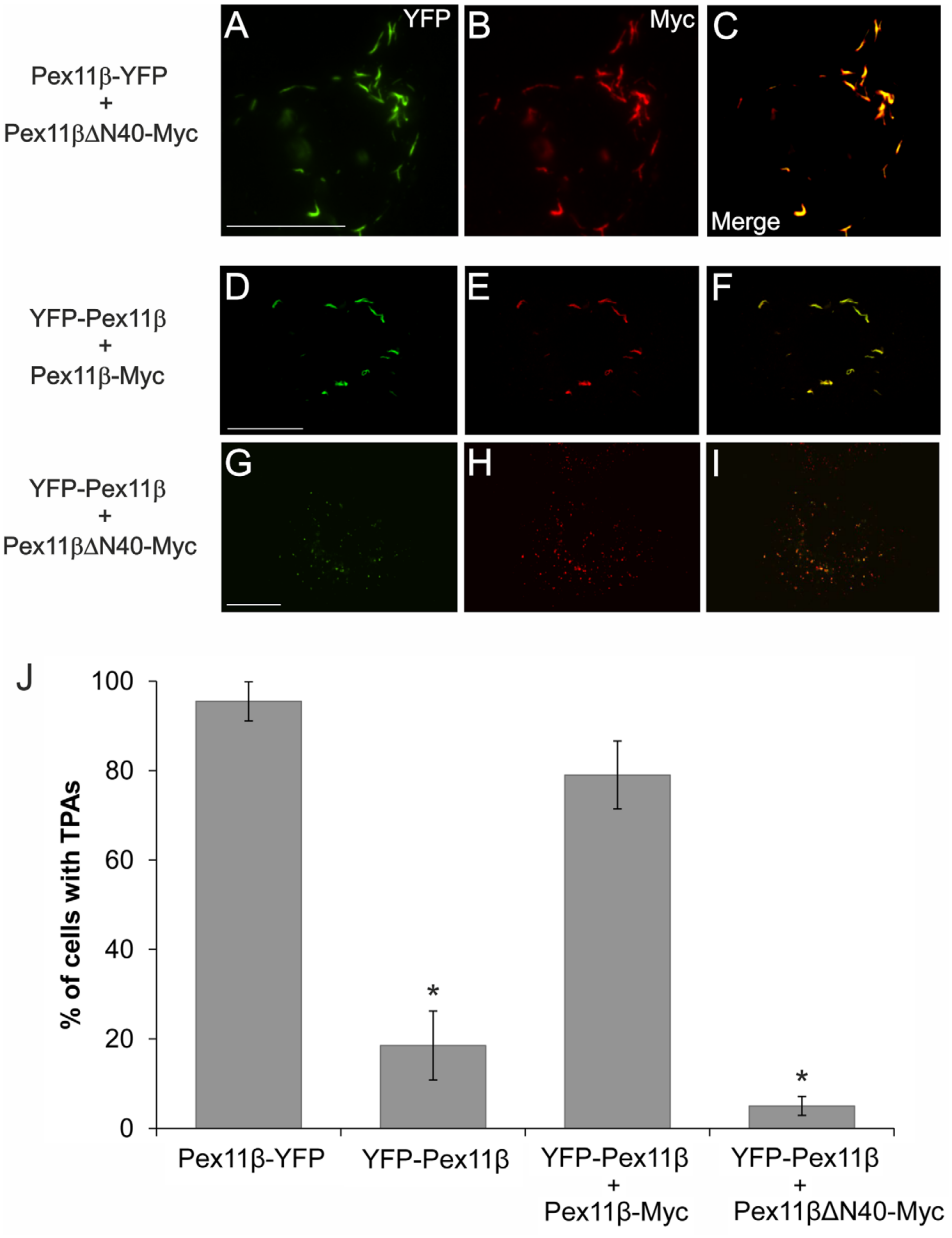
ΔN40-Pex11pβ-Myc shows altered membrane distribution within tubular peroxisomal accumulations (TPAs) and fails to induce them when co-expressed with YFP-Pex11pβ. COS-7 cells were co-transfected with Pex11pβ-YFP/Pex11pβΔN40-Myc (**A–C**), YFP-Pex11pβ/Pex11pβ-Myc (**D–F**), or YFP-Pex11pβ/Pex11pβΔN40-Myc (**G–I**) and processed as described below. Note that Pex11pβ-YFP localizes to tubular membranes (**A, C**), whereas Pex11pβΔN40-Myc distributes over both tubular and spherical membrane domains (**B, C**). Note that N-terminally tagged YFP-Pex11pβ only induces TPAs when co-expressed with Pex11pβ-Myc (**D–F**), but not with Pex11pβΔN40-Myc (**G–I**). (**J**) Quantitative evaluation of TPA formation in cells expressing Pex11pβ-YFP (a strong inducer of TPAs), YFP-Pex11pβ or co-expressing YFP-Pex11pβ/Pex11pβ-Myc or YFP-Pex11pβ/Pex11pβΔN40-Myc. Cells were fixed after 24 h, stained for immunofluorescence with anti-Myc antibodies and analyzed. Data are from 3–4 independent experiments and are presented as means ± S.D. (*p<0.01). Bars, 20 µm.

We previously described the application of a C-terminally tagged Pex11pβ-YFP construct as a novel tool to further dissect peroxisomal growth and division [14]. Pex11pβ-YFP expression resulted in the formation of tubular pre-peroxisomal membrane compartments (named TPAs), but inhibited subsequent division of the elongated peroxisomes (**Fig. 6**). The TPAs were composed out of globular membrane domains (representing mature peroxisomes) and tubular membrane extensions (forming out of the globular peroxisomes). Interestingly, peroxisomal membrane and matrix proteins distributed differently to the domains. Pex11pβ-YFP primarily localized to the tubular membrane domains; co-expression of Pex11pβ-Myc resulted in complete co-localization with Pex11pβ-YFP in the tubular membrane domains, but not in the globular ones suggesting interaction and retention of both proteins [14]. We now co-expressed Pex11pβ-YFP and the N-terminally truncated Pex11pβΔN40-Myc (**Fig. 6A–C**). Interestingly, Pex11pβΔN40-Myc was found to localize to both the tubular and globular membrane domains, indicating that its interaction or retention properties were altered.

We also previously described that the TPA-forming effect was specific for Pex11pβ-YFP and was not obtained by expression of an N-terminally tagged YFP-Pex11pβ [14] (**Fig. 6G, J** **)**. We now discovered that co-expression of YFP-Pex11pβ and Pex11pβ-Myc induced the formation of TPAs (**Fig. 6D–F, J**). In contrast, co-expression of YFP-Pex11pβ and Pex11pβΔN40-Myc was unable to induce TPA formation (**Fig. 6G–I, J**). As homo-oligomerization of Pex11pβ has been reported [24], [25], our findings let us assume that the self-interaction of Pex11pβ appears to be a pre-requisite for membrane retention and elongation of the peroxisomal membrane and is competitively disrupted by introducing the Pex11pβΔN40-Myc variant.

### The N-terminal 40 aa of Pex11pβ Including Helix 2 are Crucial for Dimer Formation

**Figure 7:**
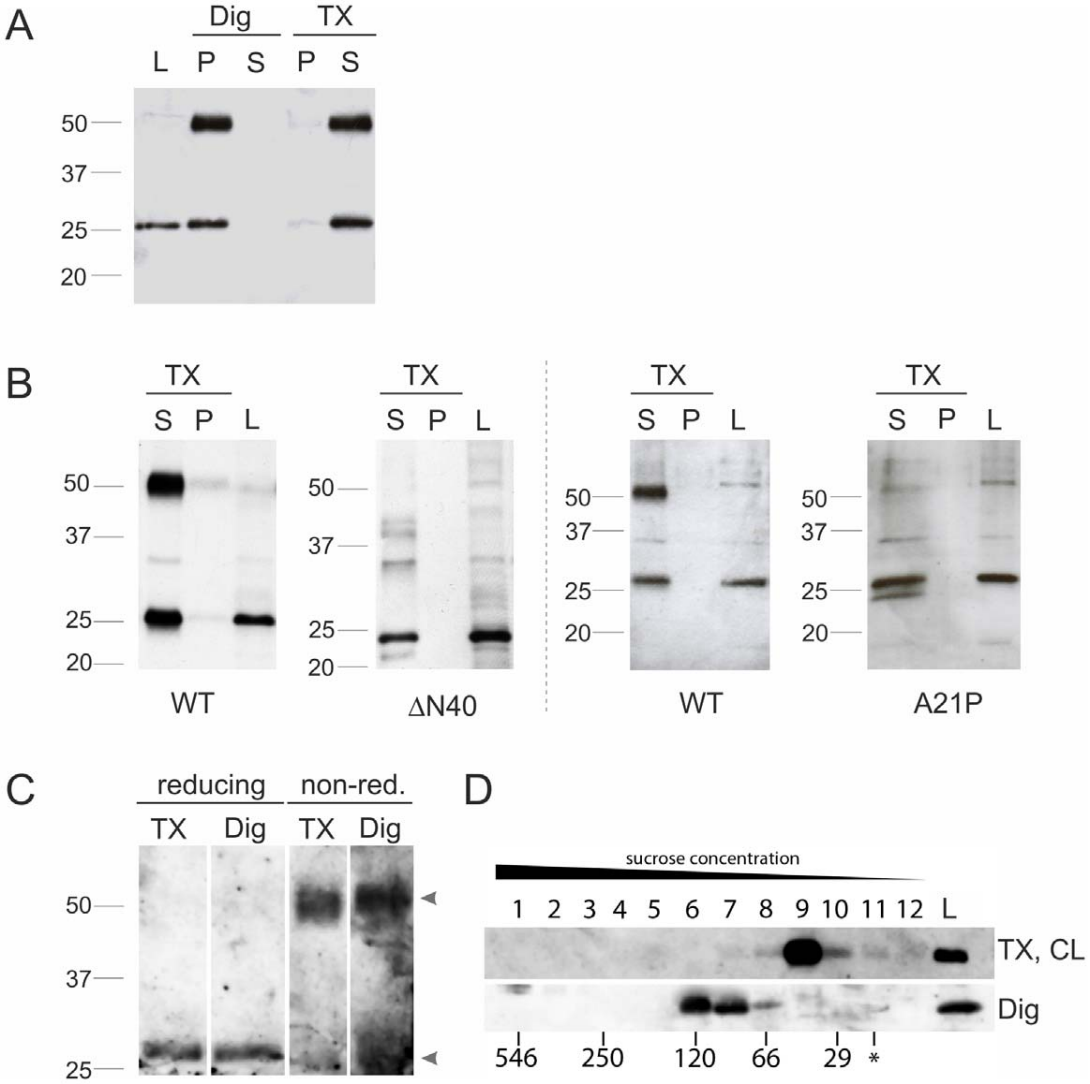
An intact Helix 2 within the first 40 N-terminal aa of Pex11pβ influences dimer formation. COS-7 cells expressing Pex11pβ-Myc (WT) (**A, B**), Pex11pβΔN40-Myc (**B**), or Pex11pβ-Myc^A21P^ (**B**) were fixed with 4% para-formaldehyde 24 h after transfection and subjected to postfixation Triton X-100 (TX) or digitonin (Dig) extraction. Equal amounts of protein from supernatants (S) (TX-extracts), remaining cell pellets (P) and untreated lysates (L) were separated by 10% SDS-PAGE and immunoblotted using anti-Myc. Note that Pex11pβ-Myc is extracted by postfixation Triton X-100 treatment but not by digitonin (**A**). (**C**) **Crosslinking of Pex11pβ-Myc with DSP.** COS-7 cells expressing Pex11pβ-Myc were cross-linked with DSP and either lysed with 1% Triton X-100 or 1% digitonin. Equal protein amounts of the lysates were separated by reducing and non-reducing (non-red.) SDS-PAGE and immunoblotted using anti-Myc. Arrowheads highlight monomeric and dimeric forms of Pex11pβ-Myc. (**D**) **Migration of Pex11pβ in native sucrose gradients**. COS-7 cells expressing Pex11pβ-Myc were either lysed in buffer containing 1% Triton X-100 (after cross-linking with DSP) (TX, CL) or in buffer containing 1% digitonin (without cross-linking) (Dig). Cell lysates were applied on top of each gradient (*), separated by sucrose density gradient ultracentrifugation (10–47%) into 12 fractions and analyzed by immunoblotting using anti-Myc. A gradient with a molecular mass marker was run in parallel for size calibration; correspondent masses are indicated at the bottom. Note the difference in the molecular mass of Pex11pβ complexes indicating different oligomerization states depending on the detergent used.

To verify this assumption, we exploited our finding that Pex11pβ (but not Pex11pα or Pex11pγ) can be extracted from peroxisomal membranes after postfixation Triton X-100 treatment [34] (**Fig. 1A**). COS-7 cells were transfected with Pex11pβ-Myc, Pex11pβΔN40-Myc, or Pex11pβ-Myc^A21P^. After 24 hours, when peroxisome elongation is maximally promoted, cells were fixed with 4% para-formaldehyde and treated with 0.2% Triton X-100 (or digitonin; **Fig. 7A**). Proteins of the detergent extract were precipitated; equal amounts were separated by SDS-PAGE and immunoblotted with anti-Myc antibodies (**Fig. 7A, B**). Interestingly, in the case of Pex11pβ-Myc, two protein bands with molecular masses of about 28 kDa and 56 kDa were detected. The 28 kDa band represents monomeric Pex11pβ-Myc, which is also detected in cell lysates (L; no fixation). The 56 kDa band likely presents a homo-dimer, which is preserved by para-formaldehyde fixation and cross-linking. Both bands are also detected after treatment of fixed cells with digitonin (**Fig. 7A**), but only in the pellet fraction further confirming that postfixation digitonin treatment does not extract Pex11pβ-Myc from peroxisomal membranes (**see** **Fig. 1**). Pex11pβΔN40-Myc, however, is only detected as a monomer in both lysates and extracts after para-formaldehyde cross-linking. Similarly, after extraction of Pex11pβ-Myc^A21P^_,_ the 56 kDa band is only faintly visible (**Fig. 7B**). Interestingly, two monomeric bands (at 28 kDa and approx. 24 kDa) were detected. The faster running band likely represents an altered conformation of the monomer, which resists unfolding in SDS-PAGE due to fixation. These findings strongly support that the Helix 2 within the first 40 aa of Pex11pβ participates in homo-dimer formation.

### Pex11pβ Complexes are Triton X-100 Sensitive

Our postfixation Triton X-100 assay revealed the formation of Pex11pβ dimers, but higher ordered oligomeric structures were not detected. However, high molecular mass complexes ranging from 230–430 kDa were recently reported by induction of peroxisome proliferation with docosahexaenoic acid [44]. To investigate Pex11pβ multimerization, we performed sucrose-density gradient fractionation (**Fig. 7D**). COS-7 cells expressing Pex11pβ-Myc were treated 24 h after transfection with the membrane-permeable crosslinker dithiobis[succinimidyl propionate] (DSP) and afterwards solubilized with 1% Triton X-100 followed by ultracentrifugation in a sucrose-density gradient (**Fig. 7D**). Efficient crosslinking by DSP was confirmed by separation of cell lysates under reducing and non-reducing conditions (**Fig. 7C**). The latter conditions preserve the crosslink resulting in a 56 kDa band shift of the monomeric Pex11pβ-Myc (**Fig. 7C**). A Pex11pβ dimer was also detected after DSP crosslinking and lysis with digitonin. After gradient centrifugation, Pex11pβ sedimented with a mass of approximately 29–66 kDa, corresponding to monomeric and dimeric forms (**Fig. 7D**). It has been reported that the use of Triton X-100 interferes with the detection of Pex11pβ-Pex11pβ interactions [24]; thus a crosslinker has to be applied. However, cell lysis with 1% digitonin solubilizes Pex11pβ while preserving its self-interaction in co-immunoprecipitation studies [24]. To avoid the use of both Triton X-100 and DSP, we therefore solubilized the cells with 1% digitonin prior to sucrose density gradient centrifugation. Interestingly, this resulted in a shift towards higher molecular masses indicating oligomeric complexes under native conditions (**Fig. 7D**). Furthermore, our findings show that the properties of Pex11pβ strongly depend on the crosslinker and detergent used for membrane solubilization.

### Lipids are Required for Proper Pex11pβ-mediated Division of Peroxisomes

**Figure 8:**
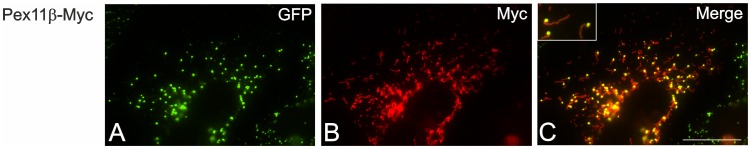
Pex11pβ-induced peroxisomal division is impaired under lipid-free culture conditions. COS-7 cells stably expressing a GFP-PTS1 fusion protein targeted to peroxisomes were incubated in lipid-free Panserin™ medium and transfected with Pex11pβ-Myc. Cells were processed for immunofluorescence using anti-Myc. Bar, 20 µm.

To investigate the requirement of lipids in Pex11pβ-mediated membrane elongation and division, we cultured COS-7 cells stably expressing a GFP-fusion protein carrying a peroxisomal targeting signal (GFP-PTS1) under lipid- and serum-free conditions. At different time points, cells transfected with Myc-Pex11pβ were analyzed by immunofluorescence microscopy using anti-Myc antibodies. Interestingly, cells cultured under lipid-free conditions revealed alterations in peroxisome morphology exhibiting enlarged, spherical organelles (**Fig. 8**). These were reminiscent to the enlarged peroxisomes observed in fibroblasts from patients with defects in peroxisomal β-oxidation enzymes [45]. In controls, the spherical peroxisomes are usually smaller, and elongated rod-shaped or tubular peroxisomes are frequently observed in the cells (see **Fig. 1**). The latter is an indication for vivid growth and multiplication of the organelles, which appears to be reduced under lipid-free conditions. Remarkably, when Myc-Pex11pβ was expressed, highly elongated membrane tubules were observed to extend from the large spherical peroxisomes, resembling “balloons” connected to a string (**Fig. 8**). This asymmetry was maintained over extended periods of time, which is unusual for Pex11pβ-induced membrane elongation and division. Furthermore, the typical membrane constrictions were very rarely observed. Interestingly, Myc-Pex11pβ was found to localize predominantly to the tubular membrane extensions and not to the globular peroxisomes (**Fig. 8**), supporting its supposed function in membrane bending and deformation. These observations further indicate that Myc-Pex11pβ can still generate and elongate membrane protrusions under lipid-free conditions. However, these do not result in proper division of the peroxisomal compartment. Thus, lipids likely contribute to the processes of membrane constriction and division. Taking into account the detergent-sensitivity of Pex11pβ, lipids may support the formation of Pex11pβ complexes within the peroxisomal membrane, and may thus modulate membrane elongation.

### The N-terminal Cysteines C18, C25 and C85 of Pex11pβ are not Essential for Membrane Elongation

**Figure 9:**
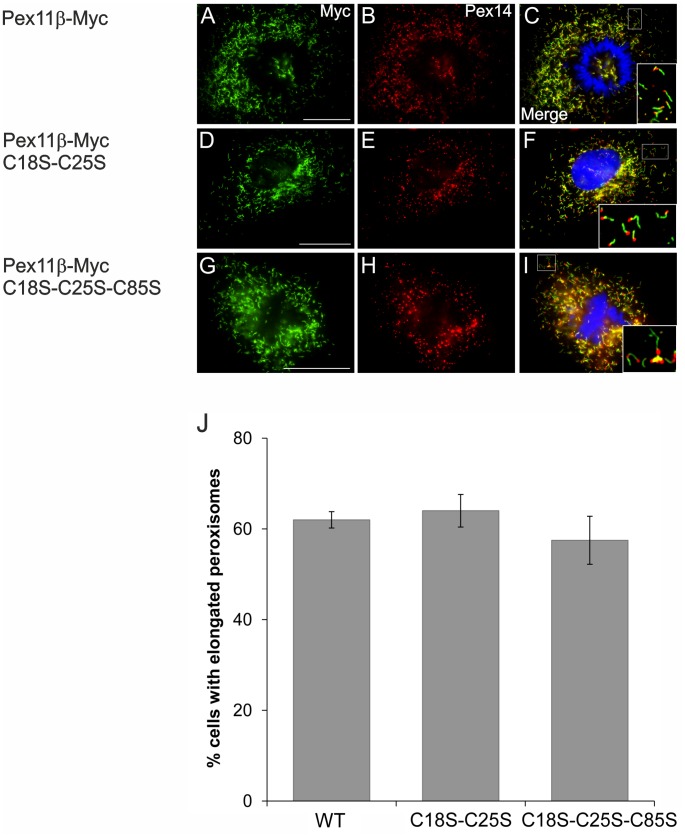
Mutations of N-terminal cysteines within Pex11pβ do not affect peroxisome membrane elongation. COS-7 cells were transfected with Pex11pβ-Myc (**A–C**), Pex11pβ-Myc^C18S-C25S^ (**D–F**) and Pex11pβ-Myc^C18S-C25S-C85S^ (**G–I**), and were processed for immunofluorescence microscopy 24 h after transfection using anti-Myc (**A, D, G**) and anti-Pex14p (**B, E, H**) antibodies. (**J**) Quantitative evaluation of peroxisome morphology. Data are from 3 independent experiments and are presented as means ± S.D. Bars, 20 µm.

Pex11pβ contains three conserved cysteines in the N-terminal domain (**Figs. S1, S2A**). To analyze if these cysteines contribute to Pex11pβ self-interaction or conformation, e.g. by the formation of intra-molecular disulfide bridges, we replaced the cysteines by serine (**Fig. S1**) and generated triple (Pex11pβ-Myc^C18S-C25S-C85S^) as well as double (Pex11pβ-Myc^C18S-C25S^) and single mutants (Pex11pβ-Myc^C18S^, Pex11pβ-Myc^C25S^, Pex11pβ-Myc^C85S^). After expression in COS-7 cells, all versions were properly targeted to peroxisomes as demonstrated by immunofluorescence microscopy using anti-Myc and anti-Pex14p antibodies (**Fig. 9A–I**) (single mutants not shown). When compared to wild-type Pex11pβ-Myc, the triple and double mutations did not interfere with the property of Pex11pβ to elongate peroxisomal membranes as confirmed by statistical evaluation (**Fig. 9J**). Similar results were obtained with the single mutants (not shown). Furthermore, even with the triple mutant, monomeric and dimeric forms of Pex11pβ were detected in immunoblots after postfixation Triton X-100 extraction (not shown). These findings indicate that the three cysteines within the N-terminus of Pex11pβ are not essential for membrane elongation.

## Discussion

Pex11 proteins in yeast, plant and animal cells contribute to the formation of peroxisomes and regulation of their abundance [6], [16], [46], [47]. Mammalian Pex11pβ has been shown to elongate and proliferate peroxisomes in conjunction with the peroxisomal division machinery and has been proposed to possess membrane remodelling/deforming properties. Its loss is embryonically lethal in knockout mice [33]. In humans, a first patient with a milder clinical phenotype but several disabilities has very recently been reported [18]. Thus, there is currently great interest in the molecular and biochemical characterization of Pex11 proteins, their mode of action and regulation of peroxisome abundance.

Here, we present a thorough analysis of the membrane topology of Pex11pβ at the peroxisomal membrane. In previous studies, we and others presented evidence that based on *in silico* studies and differential permeabilization experiments both N- and C-termini face the cytosol [21], [48], suggesting a transmembrane protein with two membrane spanning domains. However, various topologies were proposed for Pex11 proteins in different organisms [38], [39], and the related Pex11pγ was recently reported to dock on the cytosolic site of the peroxisomal membrane [20]. Furthermore, the predicted position of the first transmembrane domain (and thus the determination of the cytosolic N-terminal domain) within human Pex11pβ varies greatly, depending on the *in silico* search algorithm used, thus resulting e.g. in the designation of Pex11pβ as a tail-anchored membrane protein [49]. In the present study, we characterized a newly available Pex11pβ antibody directed against an epitope within the putative internal region (aa 110–140) to determine the topology of Pex11pβ. Using differential permeabilization, we confirmed that the epitope recognized by the Pex11pβ antibody is only accessible under conditions which permeabilize the peroxisomal membrane. Proteinase K digest of intact peroxisomes and subsequent immunoblotting with the Pex11pβ antibody revealed a protease-resistant fragment of approximately 17 kDa, which was degraded upon membrane permeabilization with either Triton X-100 or sonication. The fragment size is consistent with the localization of the first transmembrane domain between aa 90–110 (PredictProtein: aa 86–103; TMPredict: aa 96–114) [21], and the second one between aa 230–255. These data clearly demonstrate that Pex11pβ is an integral membrane protein with two transmembrane spanning domains and N- and C-termini directed towards the cytosol. The intra-peroxisomal region between the two transmembrane domains is facing the peroxisomal matrix. We cannot rigorously exclude that parts of this region may interact with the matrix site of the peroxisomal membrane, or are partially buried within the membrane. However, deletion of a glycine-rich stretch within the intra-peroxisomal region did not alter the properties of Pex11pβ to promote membrane elongation and division of peroxisomes indicating that parts of its internal region are dispensable for these functions.

Based on our results on Pex11pβ topology, we analyzed putative functional motifs in its sequence and examined their importance for the membrane-shaping properties of Pex11pβ. We focused on the cytosolic N-terminal part of the protein which contains three putative amphipathic helices [19]. Loss of most of the N-terminus (e.g. ΔN60-Pex11pβ, ΔN70-Pex11pβ) abolished membrane elongation of peroxisomes, which is consistent with recent findings reporting a loss of peroxisome proliferation after deletion of about 80 aa from the Pex11pβ N-terminus [25]. Here, we show that Helix 2 within the first 40 aa is crucial for membrane elongation and dimerization of Pex11pβ (**Figs. 5** **and** **7**). It has recently been shown that peptides matching Helix 3 can elongate liposomal structures in an *in vitro* assay suggesting a key function in peroxisome membrane elongation by docking the N-terminus to the peroxisomal membrane [19]. Our findings suggest that amphipathic Helix 3 which is still present in a ΔN40 deletion or the Helix 2-breaking mutant is not sufficient to promote peroxisome elongation or dimer formation *in situ* (**Figs. 5** **and** **7**). We propose a novel function for Helix 2 in dimer formation and retention of Pex11pβ. It is likely that both helices cooperate in Pex11pβ function.

Pex11pβ was the first peroxisomal membrane protein reported to exhibit a special distribution within the peroxisomal membrane as it was found to concentrate in constriction sites on elongated peroxisomes [21]. Furthermore, it preferentially localized to tubular membrane extensions within pre-peroxisomal membrane compartments (so called TPAs) [14]. A clear preference for tubular membrane structures was also confirmed in this study, as Pex11pβ-Myc accumulated in tubular membrane protrusions extending from enlarged peroxisomes which formed under lipid-free culture conditions (**Fig. 8**). Whereas these observations further support a role for Pex11pβ in membrane deformation and elongation, the mechanism of its targeting to and retention within these membrane domains remained unclear.

In contrast to wild type Pex11β-Myc, which was found to co-localize with Pex11pβ-YFP in the tubular membrane domains of TPAs [14], Pex11pβΔN40-Myc was not properly retained and instead distributed over the whole of the membranes (**Fig. 6**), localizing to both tubular and globular membrane domains. We suggest that the truncated version cannot be retained in the tubular domains due to impaired dimer (or complex) formation.

A self-interaction of Pex11pβ and homo-oligomerization has been suggested based on co-immunoprecipitation studies [15], [24] as well as mammalian two-hybrid assays [25]. By exploiting our previous findings that Pex11pβ-Myc (but not Pex11pα or Pex11pγ) is extracted from para-formaldehyde fixed cells by the non-ionic detergent Triton X-100 [34], we detected a monomeric and a dimeric pool of Pex11pβ-Myc after SDS-PAGE and immunoblotting (**Fig. 7**) demonstrating self-interaction and dimerization of Pex11pβ in a gel-based approach. Since the expression of Pex11pγ is barely detectable in COS-7 cells (our unpublished results), the Pex11p dimers reported under our experimental conditions are highly likely homo-dimers of Pex11pβ. Hetero-dimer formation between Pex11pβ and Pex11pα has not been described [15]. However, we cannot exclude that Pex11pβ-Pex11pγ dimers contribute to peroxisome membrane dynamics *in vivo*. Analysis of Pex11pβΔN40-Myc and Pex11pβ-Myc^A21P^ revealed predominantly monomeric pools, demonstrating that the first 40 aa and Helix 2 are crucial for dimer formation and subsequent membrane elongation. In search for higher ordered oligomeric complexes, we performed sucrose density gradient centrifugation (**Fig. 7D**). When Triton X-100 was used as a detergent in combination with the crosslinker DSP, monomeric/dimeric forms of Pex11pβ were detected, whereas higher ordered complexes were not observed (**Fig. 7D**). This is consistent with a recent study on the effect of docosahexaenoic acid (DHA) on peroxisome elongation and division [44]. However, when digitonin was used for solubilization, a shift towards higher molecular masses was observed which is in agreement with the formation of oligomeric complexes. These data reveal that Pex11pβ complexes are detergent-sensitive. This notion is confirmed by our observation that postfixation Triton X-100 treatment liberates Pex11pβ from peroxisomal membranes [34]. This unique behaviour results in impaired detection in immunofluorescence studies and is specific for Pex11pβ, but can be overcome by the use of digitonin for membrane permeabilization or the addition of a larger tag to the expressed protein, which supports crosslinking [34] (**Fig. 1**). It indicates that Pex11pβ is not strongly interacting with other proteins within the peroxisomal membrane, which renders it extractable by Triton X-100 even after fixation. Indeed, only very few interactions with other peroxins (e.g. the import receptor Pex19p or self-interactions) have been described for Pex11 proteins [16]. Furthermore, Triton X-100 interfered with the detection of Pex11pβ-Pex11pβ interactions in co-immunoprecipitation studies [24], thus requiring a crosslinking reagent [25], [44]. However, digitonin has been reported to properly solubilize Pex11pβ and to preserve its self-interactions [15], [24]. These findings strongly suggest that Pex11pβ interacts with lipids within the peroxisomal membrane, and that these lipids contribute to the formation of Pex11pβ complexes. The milder detergent digitonin likely interferes less with the Pex11pβ-lipid interactions thus preserving larger complexes. Triton X-100 on the other hand is capable of extracting Pex11pβ from fixed cells by removing and replacing its lipid-microenvironment.

In line with this, it has very recently been reported that DHA-containing phospholipids directly influence homo-oligomerization of Pex11pβ, and that incubation of acyl CoA-oxidase deficient fibroblasts with DHA resulted in hyper-oligomerization of Pex11pβ giving rise to high molecular mass complexes ranging from 230–430 kDa [44]. Intriguingly, these findings imply that Pex11pβ action on membrane elongation and thus peroxisome division is modulated by phospholipids within the peroxisomal membrane, which in turn are influenced by peroxisomal lipid metabolism such as fatty acid β-oxidation. In previous studies, we showed that the addition of polyunsaturated fatty acids (PUFAs) promotes the elongation and proliferation of peroxisomes [21]. In addition, fibroblasts from patients with defects in peroxisomal β-oxidation contain enlarged peroxisomes [45], [50]; addition of DHA, an essential PUFA and a major product of peroxisomal β-oxidation [51], however, was shown to restore peroxisome morphogenesis [44]. Here we demonstrate that cultivation of COS-7 cells in lipid-free medium promotes an enlargement of peroxisomes giving rise to large spherical organelles reminiscent of peroxisomes observed in fibroblasts from patients with AOX deficiency [52]. Expression of Pex11pβ under these conditions resulted in the formation of long membrane protrusions (**Fig. 8**) but these asymmetric structures were maintained and proper constriction and division was impaired. We conclude that lipids are required for proper peroxisome morphogenesis and division, and suggest that these processes require a subtle interplay between Pex11pβ and membrane lipids. It was proposed that phospholipids (via their bound fatty acids) directly modulate Pex11pβ oligomerization [44]. In this respect, it is possible that the concentration and type of phospholipids within the peroxisomal membrane determines and modulates Pex11pβ interaction and thus, the nature of the complexes formed. If we suggest that Pex11pβ acts like a scaffold protein, larger Pex11pβ complexes might more strongly promote peroxisome elongation than smaller ones. Certain phospholipids (as well as Triton X-100) might even directly influence Pex11pβ structure and positively or negatively regulate self-interaction. Furthermore, Pex11pβ has been reported to interact with the membrane adaptors Fis1 and Mff, which are supposed to recruit the fission GTPase DLP1 to the peroxisomal membrane [20], [25]. Fis1 and Mff are both suggested to form homo-dimers [29], [53], indicating that the formation of even larger complexes may modulate peroxisome fission. However, their preservation and detection will likely vary depending on the solubilization conditions applied. We would like to note that neither Fis1 nor Mff were found to co-migrate with the 56 kDa band of Pex11pβ-Myc in immunoblots (see **Fig. 7**), further supporting Pex11pβ-Myc dimer formation.

We as well demonstrated that the N-terminal cysteines C18, C25, and C85 are not essential for peroxisomal membrane elongation. It is unlikely that these cysteines contribute to dimer formation by covalent bonds, as Pex11pβ self-interactions in co-immunoprecipitation studies are lost in the presence of Triton X-100. In line with this, no effect on membrane elongation properties of Pex11pβ was observed in the absence of all three cysteines. It is possible that transient, intramolecular disulfide bridges exist which may stabilize Pex11pβ structure or protein interactions later on during the division process. In addition, no alterations in peroxisome elongation or division were detected when the putative N-terminal phosphorylation sites S11 and S38 within the first 40aa of Pex11pβ were altered generating putative phospho-mimicking “on” and “off” mutants (**Fig. 4**). Although phosphorylation at other putative sites within Pex11pβ is possible, we currently do not have experimental evidence that Pex11pβ is phosphorylated under our experimental conditions. The regulation of Pex11p activity by phosphorylation has recently been demonstrated for *Sc*Pex11p and *Pp*Pex11p [42], [43], however, the phosphorylation sites are not conserved among organisms. It is possible that one of the other mammalian Pex11 proteins (e.g. Pex11pα or Pex11pγ) is phosphorylated, or that other diverse regulatory mechanisms have evolved. Thus, functional and regulatory differences as well as distinct biochemical properties should be considered when investigating Pex11 isoforms or proteins from different species.

## Supporting Information

Figure S1 **Schematic view of Pex11pβ constructs used in this study.**
(TIF)

Figure S2 **(A) Overview of the location of putative amphipathic helices, transmembrane domains, potential phosphorylation sites and cysteine residues within the N-terminal portion of** ***Hs*** **Pex11pβ (aa sequence). (B) Predicted positions of the transmembrane domains of human Pex11pβ.** A variety of *in silico* screening tools were applied to determine the position of the transmembrane domains in *Hs*Pex11pβ. Based on these results, the expected size of the protein fragment between the two transmembrane domains was calculated using PeptideMass counter.
(TIF)

Figure S3 **Determination of potential phosphorylation sites within** ***Hs*** **Pex11pβ.** (**A**) Overview of multiple hits for different amino acid positions. Several online screening tools were used to determine potential phosphorylation sites in the sequence of human Pex11pβ. The various tools are plotted against the positions given. (**B**) Scheme depicting phosphorylation-sites chosen for subsequent studies. Based on the screening, several putative phosphorylation sites were selected whose location is indicated in the upper scheme (potential sites). Based on our findings regarding the topology of Pex11pβ, intra-peroxisomal sites were excluded (extraperoxisomal sites). Furthermore, based on studies regarding deletions of the N-terminus, the phosphorylation sites listed on the bottom were chosen. (**C**) Overview of conserved amino acids within Pex11pβ protein sequences across species. The putative phosphorylation-sites are depicted in red brackets. Note that the one at position S11 is highly conserved.
(TIF)

Figure S4 **Figure S4. Phospho-mimicking mutants of Pex11pβ have no effect on peroxisome elongation and division.** COS-7 cells were transfected with Pex11pβ-Myc (**A1-3, F1-3**), Pex11pβ-Myc^S11A^ (**B1-3, G1-3**), Pex11pβ-Myc^S11D^ (**C1-3, H1-3**), Pex11pβ-Myc^S38A^ (**D1-3, I1-3**) and Pex11pβ-Myc^S38D^ (**E1-3, J1-3**). Cells were fixed after 24 and 72 h, processed for immunofluorescence and labeled with antibodies directed to the Myc-epitope (**A1-J1**) and the peroxisomal marker protein Pex14p (**A2-J2**). Bars, 20 µm.
(TIF)

Table S1 **Plasmids and oligonucleotides used in this study.**
(TIF)
