# Supplementary figures and images for: Self-Interaction of Human Pex11pβ during Peroxisomal Growth and Division
Source: PLoS One. 2013 Jan 7;8(1):e53424. doi: 10.1371/journal.pone.0053424 (PMC3538539; doi:10.1371/journal.pone.0053424)

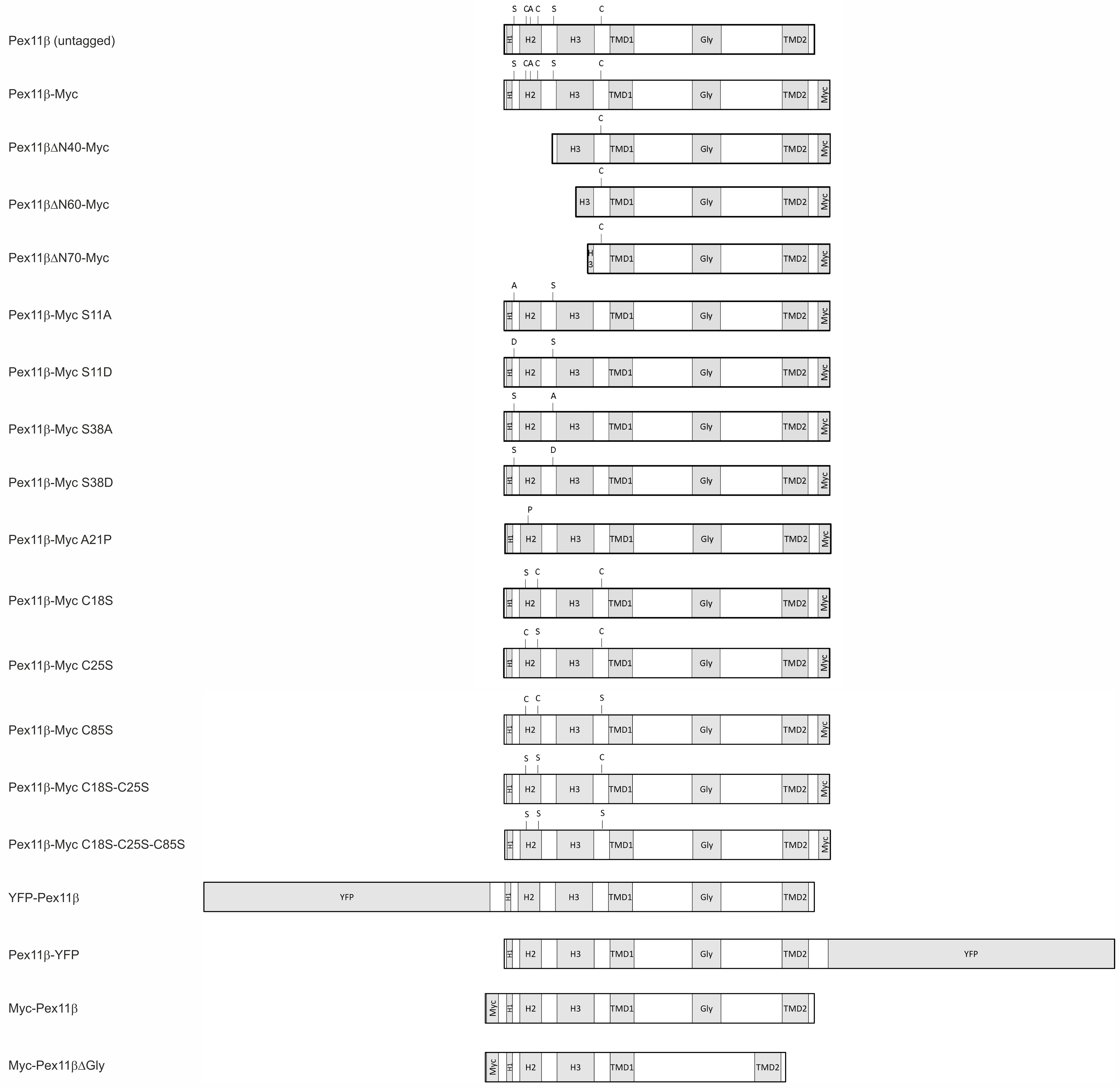

Supplement: Figure S1 — Schematic view of Pex11pβ constructs used in this study. (TIF) [file pone.0053424.s001.tif]

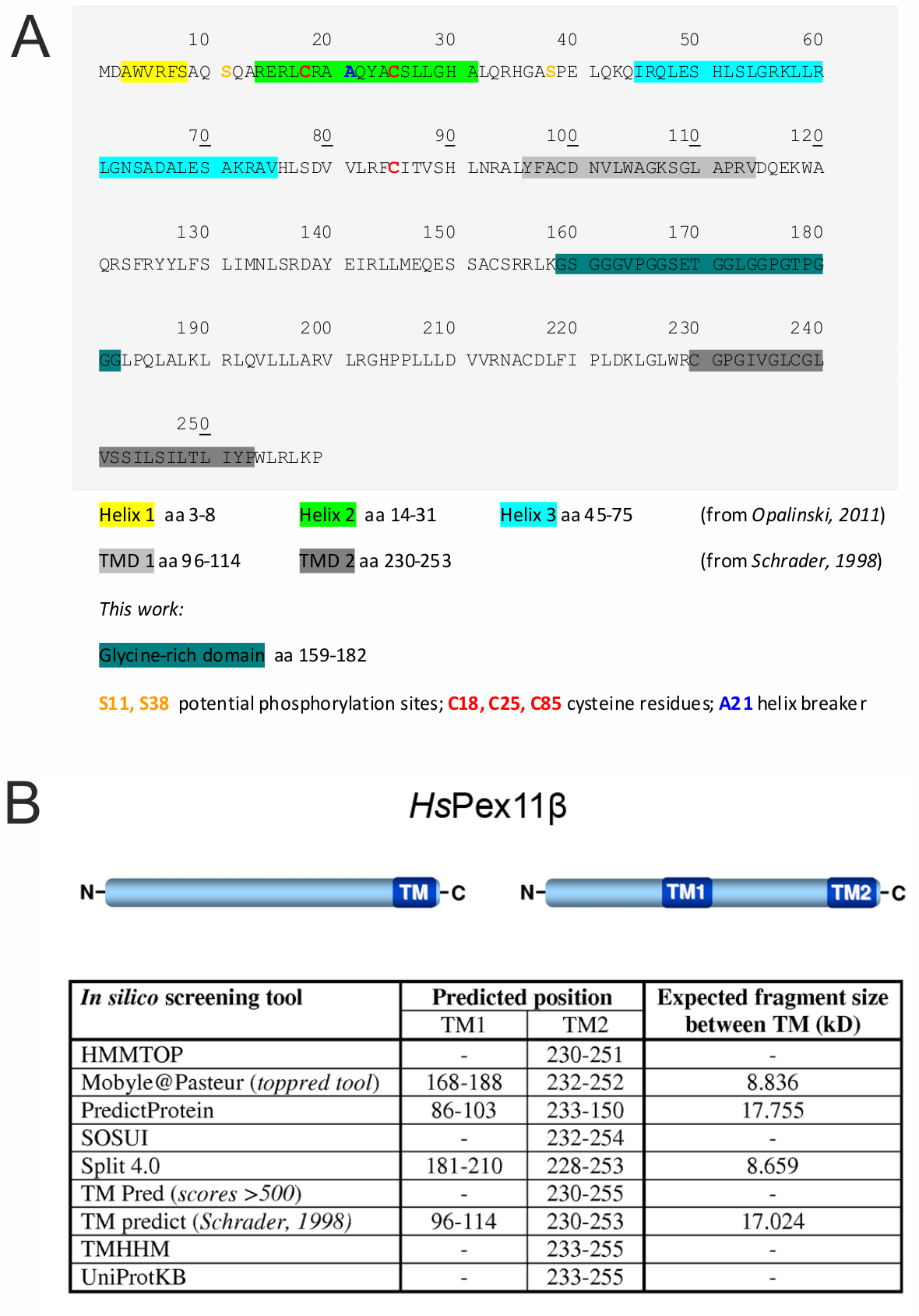

Supplement: Figure S2 — (A) Overview of the location of putative amphipathic helices, transmembrane domains, potential phosphorylation sites and cysteine residues within the N-terminal portion of Hs Pex11pβ (aa sequence). (B) Predicted positions of the transmembrane domains of human Pex11pβ. A variety of in silico screening tools were applied to determine the position of the transmembrane domains in HsPex11pβ. Based on these results, the expected size of the protein fragment between the two transmembrane domains was calculated using PeptideMass counter. (TIF) [file pone.0053424.s002.tif]

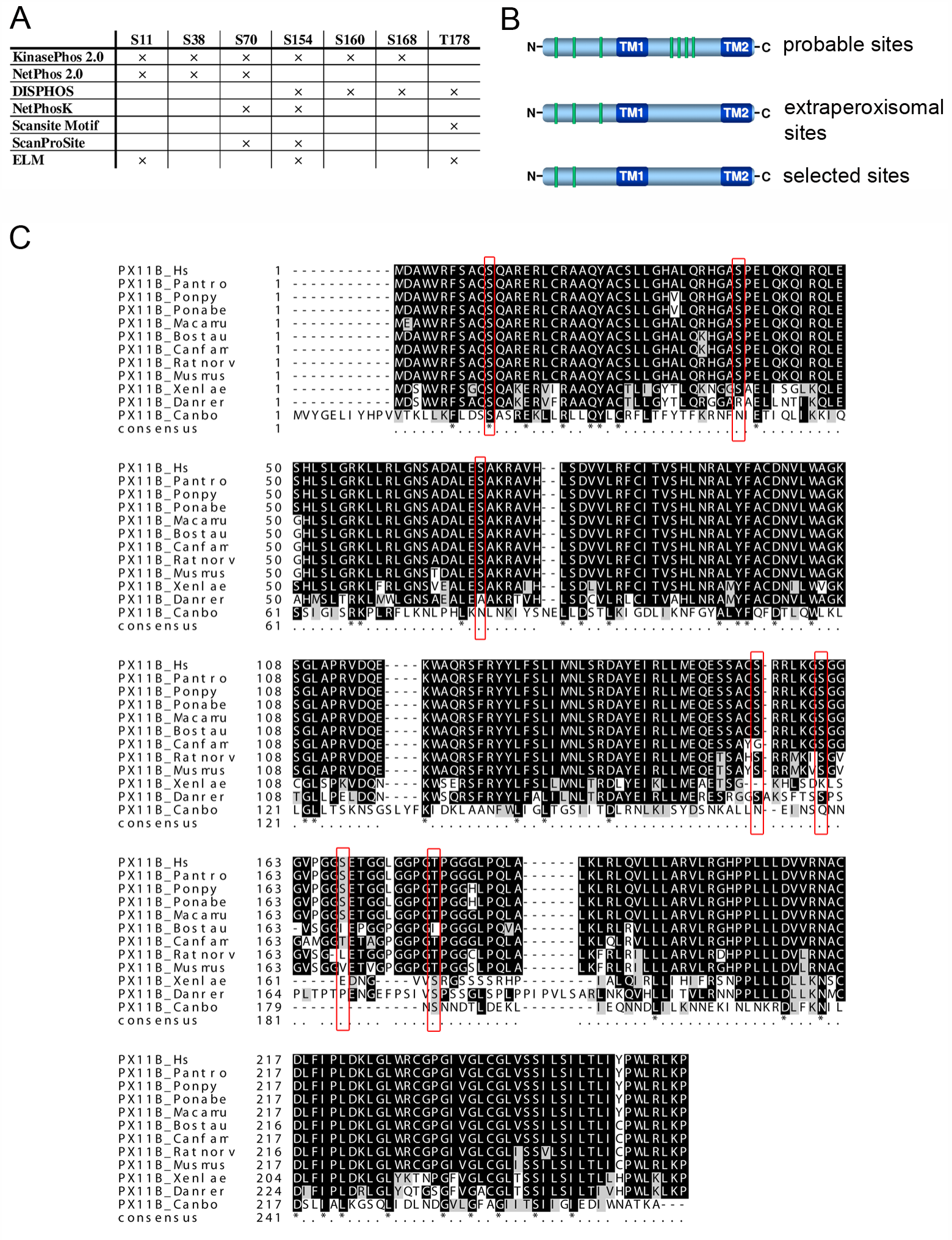

Supplement: Figure S3 — Determination of potential phosphorylation sites within Hs Pex11pβ. (A) Overview of multiple hits for different amino acid positions. Several online screening tools were used to determine potential phosphorylation sites in the sequence of human Pex11pβ. The various tools are plotted against the positions given. (B) Scheme depicting phosphorylation-sites chosen for subsequent studies. Based on the screening, several putative phosphorylation sites were selected whose location is indicated in the upper scheme (potential sites). Based on our findings regarding the topology of Pex11pβ, intra-peroxisomal sites were excluded (extraperoxisomal sites). Furthermore, based on studies regarding deletions of the N-terminus, the phosphorylation sites listed on the bottom were chosen. (C) Overview of conserved amino acids within Pex11pβ protein sequences across species. The putative phosphorylation-sites are depicted in red brackets. Note that the one at position S11 is highly conserved. (TIF) [file pone.0053424.s003.tif]

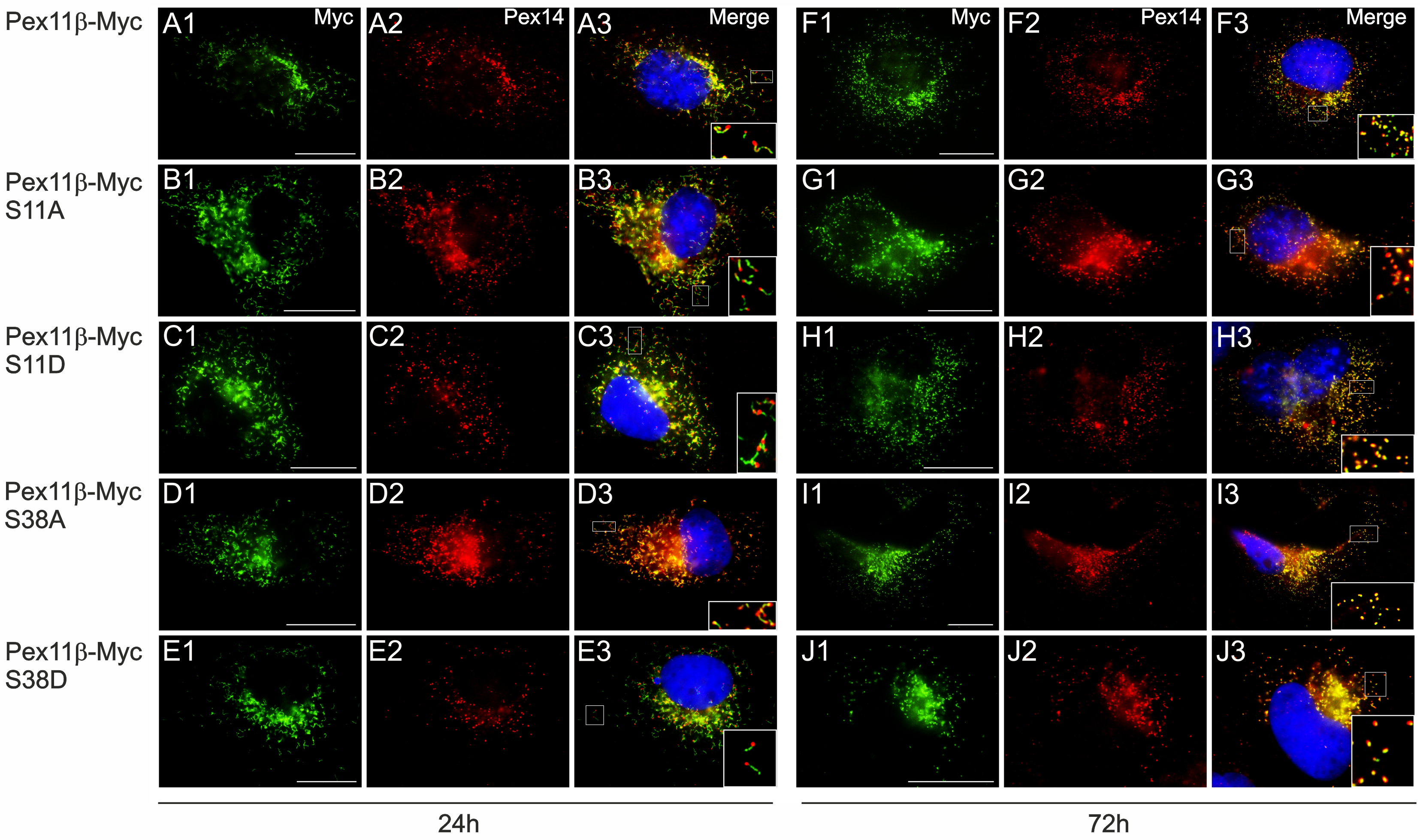

Supplement: Figure S4 — Phospho-mimicking mutants of Pex11pβ have no effect on peroxisome elongation and division. COS-7 cells were transfected with Pex11pβ-Myc (A1-3, F1-3), Pex11pβ-MycS11A (B1-3, G1-3), Pex11pβ-MycS11D (C1-3, H1-3), Pex11pβ-MycS38A (D1-3, I1-3) and Pex11pβ-MycS38D (E1-3, J1-3). Cells were fixed after 24 and 72 h, processed for immunofluorescence and labeled with antibodies directed to the Myc-epitope (A1-J1) and the peroxisomal marker protein Pex14p (A2-J2). Bars, 20 µm. (TIF) [file pone.0053424.s004.tif]

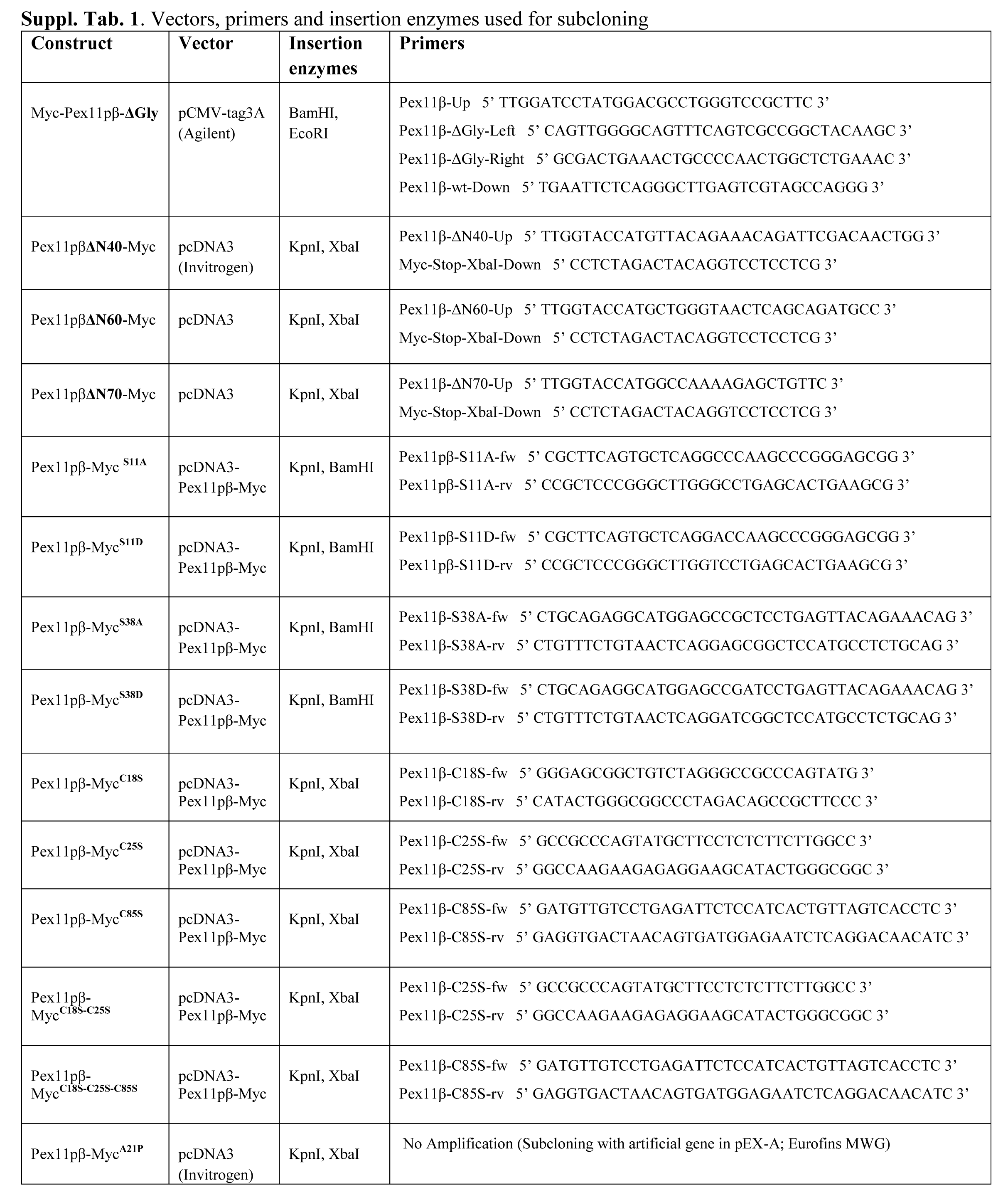

Supplement: Table S1 — Plasmids and oligonucleotides used in this study. (TIF) [file pone.0053424.s005.tif]
